# Impact of Immunoglobulin Isotype and Epitope on the Functional Properties of *Vibrio cholerae* O-Specific Polysaccharide-Specific Monoclonal Antibodies

**DOI:** 10.1128/mBio.03679-20

**Published:** 2021-04-20

**Authors:** Robert C. Kauffman, Oluwaseyi Adekunle, Hanyi Yu, Alice Cho, Lindsay E. Nyhoff, Meagan Kelly, Jason B. Harris, Taufiqur Rahman Bhuiyan, Firdausi Qadri, Stephen B. Calderwood, Richelle C. Charles, Edward T. Ryan, Jun Kong, Jens Wrammert

**Affiliations:** a Division of Infectious Disease, Department of Pediatrics, Emory University School of Medicine, Atlanta, Georgia, USA; b Department of Computer Science, Emory University, Atlanta, Georgia, USA; c Division of Infectious Diseases, Massachusetts General Hospital, Boston, Massachusetts, USA; d Department of Pediatrics, Harvard Medical School, Boston, Massachusetts, USA; e Infectious Diseases Division, International Centre for Diarrhoeal Disease Research, Dhaka, Bangladesh; f Department of Medicine, Harvard Medical School, Boston, Massachusetts, USA; g Department of Immunology and Infectious Diseases, Harvard T. H. Chan School of Public Health, Boston, Massachusetts, USA; h Department of Mathematics and Statistics, Georgia State University, Atlanta, Georgia, USA; Wadsworth Center, New York State Department of Health; University of Mississippi Medical Center

**Keywords:** immunoglobulin isotype, OSP antibody, *Vibrio cholerae*

## Abstract

Vibrio cholerae causes the severe diarrheal disease cholera. Clinical disease and current oral cholera vaccines generate antibody responses associated with protection. Immunity is thought to be largely mediated by lipopolysaccharide (LPS)-specific antibodies, primarily targeting the O-antigen. However, the properties and protective mechanism of functionally relevant antibodies have not been well defined. We previously reported on the early B cell response to cholera in a cohort of Bangladeshi patients, from which we characterized a panel of human monoclonal antibodies (MAbs) isolated from acutely induced plasmablasts. All antibodies in that previous study were expressed in an IgG1 backbone irrespective of their original isotype. To clearly determine the impact of affinity, immunoglobulin isotype and subclass on the functional properties of these MAbs, we re-engineered a subset of low- and high-affinity antibodies in different isotype and subclass immunoglobulin backbones and characterized the impact of these changes on binding, vibriocidal, agglutination, and motility inhibition activity. While the high-affinity antibodies bound similarly to O-antigen, irrespective of isotype, the low-affinity antibodies displayed significant avidity differences. Interestingly, despite exhibiting lower binding properties, variants derived from the low-affinity MAbs had comparable agglutination and motility inhibition properties to the potently binding antibodies, suggesting that how the MAb binds to the O-antigen may be critical to function. In addition, not only pentameric IgM and dimeric IgA, but also monomeric IgA, was remarkably more potent than their IgG counterparts at inhibiting motility. Finally, analyzing highly purified F(ab) versions of these antibodies, we show that LPS cross-linking is essential for motility inhibition.

## Abstract (precis)

Immunity to the severe diarrheal disease cholera is largely mediated by lipopolysaccharide (LPS)-specific antibodies. However, the properties and protective mechanism of functionally relevant antibodies have not been well defined.

## INTRODUCTION

Protective immunity to cholera is thought to be predominantly antibody mediated (1). Both clinical disease and current oral cholera vaccines (OCVs) generate responses in the serum and gastrointestinal tract that are associated with protection (2, 3). Clinical disease is thought to result in long-term, durable immunity that lasts 3 to 10 years (4, 5) and provides nearly 100% protection. In contrast, OCVs have an average two-dose efficacy of only 58% after 60 months (6). Protection is thought to be primarily mediated by lipopolysaccharide (LPS)-specific antibodies (7). Of the approximately 200 serogroups in circulation, pathogenic V. cholerae isolates are comprised of just two different serogroups, O1 and O139, which differ based upon their LPS structure (8). Importantly, protection is strictly serogroup specific, underscoring the importance of LPS-specific antibodies in conferring protective immunity (9, 10). The O1 serogroup is currently responsible for most of the 2.9 million cholera related infections and more than 100,000 deaths that occur each year (11). This serogroup is further subdivided into the Ogawa and Inaba serotypes. Although their LPS structures are nearly identical, they differ by the presence of a single methyl group on the terminal sugar moiety of the 12- to 16-subunit-long O-antigen that extends from the core oligosaccharide (12).

In mice, immunization with soluble V. cholerae O1 derived LPS has been shown to generate antibody responses that recognize the O-antigen, the core sugar, and the lipid A components of the LPS molecule (13). In contrast, V. cholerae infected humans generate LPS-specific antibody responses that almost exclusively target the O-antigen (7, 14). A significant body of evidence demonstrates that these O-antigen-specific antibody responses are strongly associated with immunity following infection (2, 15). Through a process termed immune exclusion, V. cholerae LPS-specific antibodies are thought to inhibit successful colonization of the small intestine, thus preventing the production and release of cholera toxin, the protein exotoxin responsible for the severe diarrheal symptoms associated with cholera (16, 17). The exact mechanisms that allow antibody-mediated inhibition of bacterial colonization have not been fully elucidated but likely occur through a multifactorial series of mechanisms, including bacterial agglutination (18), enchaining dividing cells (19), and inhibiting bacterial motility (20, 21).

Serum vibriocidal antibodies are associated with protective immunity and are a critical component in vaccine evaluation (22). Early serum depletion studies have suggested that vibriocidal titers are primarily derived from LPS O-antigen-specific IgM and, to a lesser extent, IgG1 and IgG3 antibodies (7, 23). Despite this, complement-mediated protection is unlikely to be a critical component of mucosal immunity to a noninvasive bacterial pathogen given the small amount of complement in the intestinal tract (24, 25). Moreover, vibriocidal titers decline to baseline levels before protective immunity wanes (26). Thus, vibriocidal antibody titers are likely an indirect marker of protection, which presumably occurs at the site of infection in the small intestine. In contrast to vibriocidal IgG and IgM serum antibodies, LPS-specific IgA in the intestinal mucosa likely plays a critical role in long-term disease protection (27). IgA is the predominant immunoglobulin isotype in the intestine, where it persists in a J-chain-stabilized, dimeric form (28). IgA is also actively transported from the lamina propria, through the epithelial cell layer into the lumen, where it interacts with the mucus layer (reviewed in reference 29). Importantly, cholera infection and vaccination have been shown to induce significant increases in LPS-specific duodenal IgA (27, 30). Moreover, infection induces a significant number of LPS-specific IgA secreting lamina propria plasma cells. These responses peak 30 days after infection and can persist through at least day 180 (27). However, mucosal LPS-specific IgA titers wane before protection is lost (27), suggesting that not only mucosal plasma cells but perhaps also memory B cells are a key component of long-term cholera immunity. The importance of memory B cells (MBCs) is supported by studies showing that circulating LPS-specific MBCs are associated with a decreased risk of infection in household contacts of cholera patients (31) and that LPS-specific MBCs remain elevated for at least 1 year after infection, which is longer than traditional measures of antibody-mediated immunity by enzyme-linked immunosorbent assay (ELISA) and vibriocidal titers (32). Together, these findings suggest that a combination of preexisting LPS-specific antibody-secreting plasma cells and rapid anamnestic responses derived from MBCs are critical to protection in the gut mucosa.

To better understand how cholera induces immunity, we recently reported the use of single-cell monoclonal antibody (MAb) expression cloning to generate and characterize the properties of infection-induced plasmablast responses in patients from Dhaka, Bangladesh, an area where cholera is endemic (14). Plasmablasts emerging shortly after human infection or vaccination are enriched for antigen-specific antibody-secreting cells (33). Therefore, plasmablasts represent a valuable source of antibodies that reflect the properties and natural history of both preexisting and novel responses. Repertoire analysis of cholera-induced plasmablasts demonstrated that clonally expanded populations were often enriched for LPS-specific cells (14). In addition, many of the antibodies were highly mutated and bound more potently to the O1-Inaba serotype that had not been in circulation for the last 5 years, suggesting that these cells derived from recall responses to a previously encountered polysaccharide antigen. These MAbs also demonstrated a wide range of binding potency toward the LPS molecule (14). The antibodies in the aforementioned study bound with equal affinity to purified LPS and OSP-core conjugated to bovine serum albumin (BSA), indicating that they do not target the lipid A component of LPS (14). In addition, their binding, vibriocidal, and agglutination properties were determined for both the Ogawa and Inaba O1 serotypes. It is possible that the binding site of some MAbs may overlap the core domain; however, the four MAbs analyzed in this study demonstrated serotype selectivity in these assays. Given that the serotypes are distinguished by the structure of the O-antigen, we hypothesize that they are primarily specific for the O-specific polysaccharide moiety of LPS. Finally, our analysis of Ig isotype demonstrated that the majority of LPS-specific antibodies were primarily derived from IgM- and IgA-secreting plasmablasts (14); however, to facilitate their examination, the initial characterizations of these antibodies were executed in a human IgG1 background regardless of the original isotype (14).

Despite the importance of LPS-specific antibodies in mediating protective immunity, the impact of Ig isotype and subclass on the functional qualities of these antibodies has not been rigorously characterized. Moreover, the exact mechanisms by which LPS-specific antibodies confer protection remain to be fully elucidated. Given the difference in Ig distribution in blood and mucosal tissue, we sought in this study to assess the impact of Ig isotype and subclass on the functional properties of O-antigen-specific antibodies in the context of an identical antigen binding site over a range of different binding potencies. To that end, we determined that modifications to the immunoglobulin isotype and subclass, which presumably increase antibody avidity, markedly enhanced the agglutination and motility inhibition functional properties of the antibodies. This demonstrates a critical role of O-antigen cross-linking in mediating these processes, a finding that was further supported by analyses of F(ab) fragments generated from this panel. Intriguingly, we also observed that MAbs with a lower binding strength generally had comparable or modestly superior motility inhibition properties to the high-binding MAbs, suggesting that the unique steric interaction between an antibody and the O-antigen can strongly influence functional potency. Therefore, the findings presented here inform future rational vaccine and therapeutic MAb development approaches, by providing insight into the impact of Ig isotype, subclass, and epitope on the functional potency of LPS O-antigen-specific antibodies.

## RESULTS

### Generation of a panel of immunoglobulin isotype and subclass variants of LPS O-antigen binding MAbs with a range of binding potencies.

**FIG 1:**
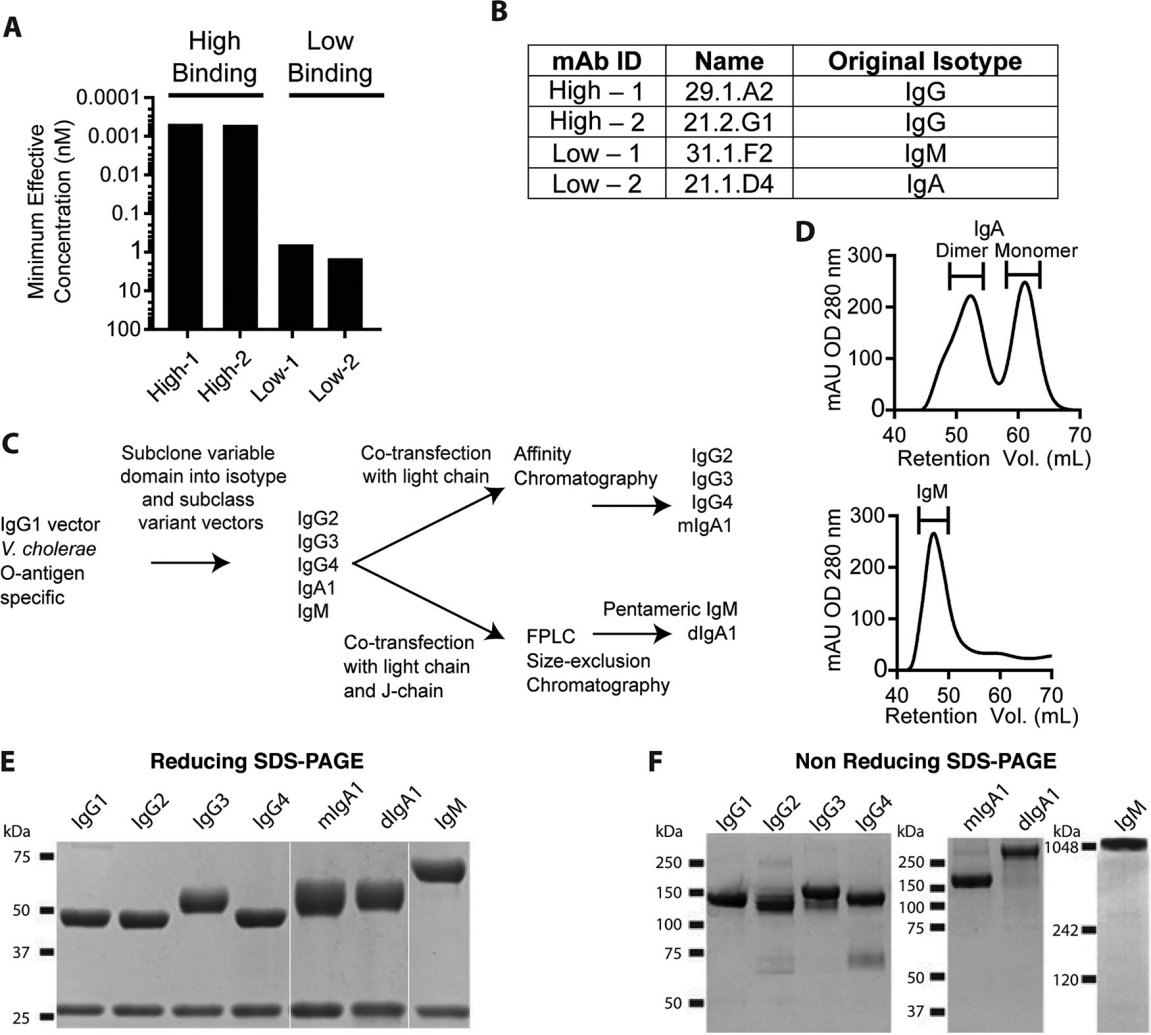
Generation of immunoglobulin isotype and subclass variants from LPS O-antigen-specific MAbs with differential binding strengths. (A) IgG1 variant binding to O1-Ogawa O-antigen was determined by ELISA. Values represent MAb concentration with signal three times background. (B) Table detailing the original nomenclature and sorted cell isotype of the parent antibody previously reported (see reference 14). (C) Flow chart describing the generation of antibody isotype and subclass variants. (D) Representative 280-nm sample absorbance chromatograms of IgM and IgA purification during SEC. Brackets denote the collected fractions. (E) Reducing SDS-PAGE analysis of isotype and subclass variants of MAb High-1. Heavy (top band) and light (bottom band) chains were resolved using Mini-Protean TGX 10% gels (Bio-Rad). (F) Nonreducing SDS-PAGE analysis of immunoglobulin variants derived from MAb High-1. IgG and IgA antibodies were resolved using NuPAGE 4 to 12% Bis-Tris Gels with MOPS-SDS running buffer. IgM samples were resolved on a Native PAGE 3 to 12% Bis-Tris gel with Tris-acetate-SDS running buffer. The data are representative of two or more independent experiments.

To define the impact of isotype and subclass on the functional qualities of LPS O-antigen-specific antibodies over a range of binding potencies, we selected a subset of antibodies identified in a previous study of Bangladeshi cholera patients (14). This study included 138 MAbs generated from single-cell expression cloning of infection-induced plasmablasts. Of these, 24 MAbs were LPS-specific and presumably recognized the O-specific polysaccharide (OSP) moiety of LPS. For the initial analysis of these cells, MAbs were expressed in an IgG1 backbone as previously described (33, 34). To further understand the impact of isotype and subclass on the binding and functional qualities of these LPS-specific antibodies, we selected two MAbs that bound the O-antigen with apparent high-affinity and two that bound with low-affinity. Figure 1A shows the binding qualities by ELISA of these four antibodies expressed as human IgG1, demonstrating a 100- to 1,000-fold range in binding activity against V. cholerae O1-Ogawa O-antigen, which had been conjugated to BSA.

The heavy-chain binding sites (VDJ fragment) from these four MAbs were subcloned into a panel of vectors with different constant regions and then expressed as monomeric human IgG1, IgG2, IgG3, IgG4, IgA1 (mIgA1), dimeric IgA1 (dIgA1), and pentameric IgM. For simplicity, the MAbs are named here based on their binding potency, with the original nomenclature, and plasmablast antibody isotype shown in Fig. 1B. As summarized in the flow chart in Fig. 1C, after variable domain subcloning, monomeric MAbs were produced by transient transfection of heavy and light chains. For the expression of dIgA and pentameric IgM, transfections were performed using a third J-chain expression vector. IgA transfections containing the J chain generated a mixed population of monomeric and dimeric MAbs, which were separated by size-exclusion chromatography (SEC) after initial affinity column purification (Fig. 1D, left). IgM was purified by SEC (Fig. 1D, right), yielding preparations with approximately 90% purity. Figure 1E shows a representative gel where MAb variants have the same size light chain (bottom band) but differently sized heavy chains (top band). Using this type of analysis, the protein content of these preparations was normalized based on densitometry analysis of the light chain band following reducing SDS-PAGE. Throughout the course of functional studies, MAbs were evaluated by nonreducing SDS-PAGE to confirm structural integrity. A representative nonreducing gel showing MAbs of the expected size is shown in Fig. 1F.

### Isotype variants of high-binding MAbs have comparable binding properties but different functional characteristics.

**FIG 2:**
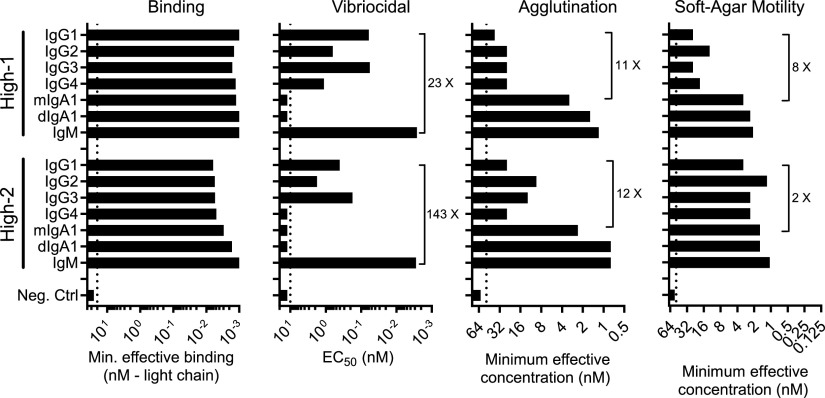
Isotype does not markedly impact the binding properties of high-binding MAbs but differentially affects their functional potency. Quantitative analysis of binding, vibriocidal activity, agglutination potency, and motility inhibition for immunoglobulin variants derived from the two high-binding MAbs. For all analyses, an influenza HA-specific human IgG1 antibody (EM4C04) was used as a negative control. Dotted lines indicate the highest tested concentration for each assay (binding = 20 nM; vibriocidal = 10 nM, agglutination = 50 nM, and motility = 50 nM). Vibriocidal values reflect the EC_50_ after baseline subtracting data generated using paired heat-inactivated complement controls. The numbers next to the brackets indicate the fold increase of the selected isotype over the corresponding IgG1 values. Values represent the means of duplicate measurements in one experiment and are representative of results from two independent experiments.

We focused our initial efforts on the two high-affinity antibodies because changing the isotype and valency of the MAb did not markedly impact their antigen binding properties. As shown in Fig. 2, changing the constant domain did not affect the binding properties of the MAb High-1. However, increasing the avidity of MAb High-2 by expression either as dimeric IgA1 or pentameric IgM did appear to modestly enhance binding potency. Since changing the constant domain had minimal affect on binding, we could directly evaluate the effect of immunoglobulin isotype and subclass on the functional properties of these MAbs. To evaluate function, we tested this panel of antibodies in vibriocidal, agglutination, and motility inhibition assays. Representative experimental data from these functional assays are shown in Fig. S1 in the supplemental material.

10.1128/mBio.03679-20.1 FIG S1 Representative experimental data from binding and functional analyses. (A to D) Representative functional assays for selected variants of MAb High-1. (A) Binding to Ogawa O antigen was determined by ELISA. The dotted line marks the absorbance value equivalent to three times the background signal. This threshold was used to quantify binding strength in all reported ELISAs. (B) Vibriocidal assay EC_50_ values were determined as the concentration of antibody (*x* axis) capable of effecting a 50% reduction in bacterial growth relative to no-treatment controls. Higher values on the *y* axis correspond to increased culture turbidity, as measured from the UV absorbance at 595 nm. The dotted line signifies the EC_50_ threshold. (C) Agglutination potency was determined using a microtiter plate-based agglutination assay. The reported minimal effective concentration (red squares) was determined as the lowest concentration of antibody capable of visibly agglutinating approximately 1 × 10^8^ CFU of unfixed bacteria. This is evident by the absence of a defined “button” of settled bacteria as seen in a no-antibody control well (–). (D) Motility inhibition was determined using a soft-agar motility assay in which serially diluted MAbs were infused in 0.3% LB-agar. The reported minimal effective concentration (red square) was determined as the lowest concentration of antibody capable of visibly retarding bacterial migration relative to the no treatment control well (–). Red lines denote the leading front of bacterial migration. Download FIG S1, PDF file, 0.5 MB. Copyright © 2021 Kauffman et al. 2021 Kauffman et al. https://creativecommons.org/licenses/by/4.0/ This content is distributed under the terms of the Creative Commons Attribution 4.0 International license.

Vibriocidal titers have been strongly correlated with protective immunity. Our analyses of the two high-binding MAbs demonstrated that the IgM variant was the most potent isotype. In fact, relative to IgG1, IgM derived from MAbs High-1 and MAb High-2 was 20 and 150 times more potent, respectively. Among the IgG subclass variants, IgG1 and IgG3 had the most vibriocidal activity. This is in agreement with their ability to activate the classical complement pathway (35). While IgG4 does not activate the classical complement pathway (35), we found that this variant had modest vibriocidal activity. This may reflect high epitope density enhancement of the alternative pathway (36). For these vibriocidal analyses, data were background subtracted from experiments performed in parallel with heat-inactivated complement. This is important since, for each MAb, we observed an apparent low-level complement-independent inhibition of bacterial outgrowth in the vibriocidal assay possibly due to antibody-mediated agglutination or enchaining of dividing cells. This effect was strongest for dimeric IgA1 and IgM antibodies. A representative example of data collected from MAb High-1 is shown in Fig. S2, illustrating the magnitude of vibriocidal 50% effective concentration (EC_50_) values derived from assays using either fresh or heat-inactivated complement.

10.1128/mBio.03679-20.2 FIG S2 Antibodies demonstrate complement-independent inhibition of bacterial outgrowth in standard V. cholerae vibriocidal assays. EC_50_ values were derived from vibriocidal analyses of MAb High-1 variants in which experiments were performed with either fresh 5% guinea pig complement (black bars) or 5% heat-inactivated guinea pig complement (white bars). Complement was inactivated at 56°C for 30 min. EM4C04-IgG1 was used as a negative control. The dotted line at 20 nM denotes the lowest concentration tested in heat-inactivated complement samples. Values represent the mean of duplicate measurements in one experiment and are representative of two independent experiments. Download FIG S2, PDF file, 0.07 MB. Copyright © 2021 Kauffman et al. 2021 Kauffman et al. https://creativecommons.org/licenses/by/4.0/ This content is distributed under the terms of the Creative Commons Attribution 4.0 International license.

Next, we investigated the effect of isotype and subclass on bacterial agglutination. After ingestion of V. cholerae, agglutination may enhance immune exclusion and, through enchaining, inhibit bacterial growth. As seen in Fig. 2, changing the IgG subclass had minimal effect on the agglutination properties. In contrast, dimeric IgA1 and IgM, resulted in a 10- to 30-fold increase in agglutination potency. Surprisingly, despite having comparable binding properties, monomeric IgA also displayed dramatically enhanced agglutination activity with potencies >10 times greater than that observed for the monomeric IgG1 variant for each MAb (Fig. 2).

Inhibition of bacterial motility is thought to be a major mechanism of antibody mediated protection (21, 37). Thus, as shown in Fig. 2, we used a soft-agar migration assay to quantify the effect of our antibody variant panel on motility. We found that changing the IgG subclass of MAb High-1 had a minimal effect on motility inhibition, which is similar to our findings with agglutination. In contrast, IgM and dimeric IgA1 variants were 5- to 10-fold more potent than IgG1. Surprisingly, monomeric IgA1 was 8-fold more potent than IgG1; however, this difference was less pronounced for MAb High-2 whose baseline IgG1 motility inhibition titer was higher than MAb High-1. Taken together, these data demonstrate that although the immunoglobulin isotype and subclass has a minimal impact on the binding strength of these two MAbs, their functional potency can be dramatically impacted by changes in their respective antibody constant region and antigen binding site valency.

### Binding potency and avidity do not directly dictate effectiveness.

**FIG 3:**
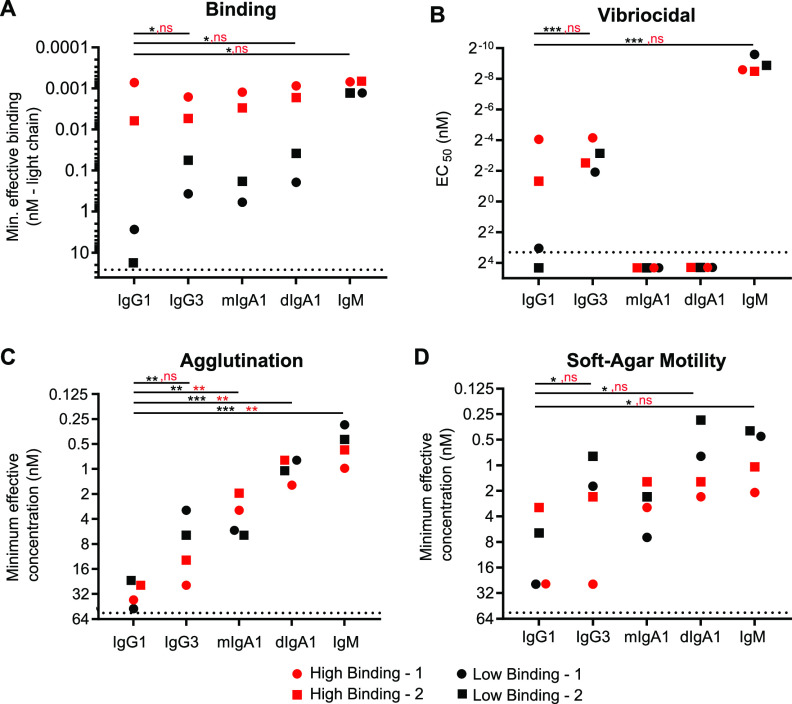
Low-binding MAb variants displayed functional properties comparable to high-binding MAbs, and O-antigen binding was markedly affected by isotype and subclass. (A) Variant binding to O1-Ogawa O-antigen was determined by ELISA. Values represent MAb concentration with signal three times background. (B) Vibriocidal assay EC_50_ values of select antibodies after background subtraction of inactivated complement controls. (C) Values represent the minimum effective MAb concentration that agglutinated bacteria in a microtiter agglutination assay. (D) Values represent the minimum effective MAb concentration that inhibited bacterial migration relative to a no antibody control in a soft-agar motility inhibition assay. The dotted line marks the highest concentration of antibody evaluated (A = 20 nM, B = 10 nM, C = 50 nM, D = 50 nM). Values represent the means of duplicate measurements and are representative of results from two independent experiments. Significance between bracketed samples was determined using a two-way ANOVA. Significance for low affinity MAbs is indicated in black, and significance for high-affinity MAbs is indicated in red. Significance values are indicated by asterisks (*, *P* < 0.05; **, *P* < 0.005; ***, *P* < 0.0005; ****, *P* < 0.00005).

Following our initial binding and functional analyses of the high-affinity antibody isoforms, we next examined a comparable panel generated from two antibodies that displayed poor O-antigen binding strength when expressed as IgG1. Binding analyses demonstrated a profound effect of immunoglobulin isotype, subclass, and valency on the binding strength of these two antibodies, which is in stark contrast to what we observed for the two high-binding antibodies (Fig. 3A). Despite having an identical antigen binding domain, antibodies expressed as IgG3 had a 10- to 100-fold increase in binding potency relative to the IgG1 variant. The monomeric and dimeric IgA versions of these MAbs also displayed enhanced binding strength, at levels comparable to IgG3; the dimeric version of each IgA1 MAb exhibited ∼4-fold higher potency than its monomeric IgA1 counterpart, presumably due its higher avidity (Fig. 3A). IgM variants had the highest binding potency displaying a striking 1,000- to 10,000-fold increase in the minimal effective binding concentration relative to IgG1. Interestingly, both low- and high-affinity antibodies had equally strong binding potencies when expressed as IgM despite their dramatically different binding potencies when expressed as IgG1.

The vibriocidal activity of an antibody is highly dependent on both its capacity to bind antigen and the ability of its constant domain to activate specific complement proteins. Consequently, for the two low-binding-strength antibodies, assessing the direct contribution of the constant domain to vibriocidal activity is confounded by the fact that changing the isotype and subclass also markedly affected the binding properties (Fig. 3A). Given that context, while the vibriocidal capacity of the IgG1 variants was lower than that of the high-binding antibodies, the IgM and IgG3 variants were similar (Fig. 3B). Lastly, we found IgM to be the most potent isoform displaying up to 10,000 times higher vibriocidal activity than IgG1 (see Fig. S3), further underscoring the importance of this isotype to the overall vibriocidal titer.

10.1128/mBio.03679-20.3 FIG S3 IgM variants have markedly enhanced vibriocidal capacity for all antibodies. Vibriocidal assay EC_50_ values of antibodies after background subtraction of heat-inactivated complement controls. Brackets above bars represent the fold increase in potency between IgG1 and IgM and between IgG1 and IgG3 MAbs. EM4C04-IgG1 was used as a negative control. The dotted line at 10 nM denotes the lowest concentration tested in experiments containing active complement. Values represent the mean of duplicate measurements in one experiment and are representative of two independent experiments. Download FIG S3, PDF file, 0.2 MB. Copyright © 2021 Kauffman et al. 2021 Kauffman et al. https://creativecommons.org/licenses/by/4.0/ This content is distributed under the terms of the Creative Commons Attribution 4.0 International license.

Despite the significant differences in binding potency, the agglutination activity of both low-binding MAb Ig variants (Fig. 3C, black symbols) was surprisingly similar to that of the two high-binding MAbs (Fig. 3C, red symbols). For example, the IgG3 variant of MAb Low-1 has a binding titer that is 1,000-fold lower than MAb High-1 (IgG3) and 300-fold lower than MAb High-2 (IgG3). Nevertheless, we observed that, relative to MAb High-1, it was more effective at agglutinating bacteria. Analyses of mIgA1 were more similar, with the high-binding MAbs displaying 2- to 4-fold-enhanced agglutinating potential; however, even this modest enhancement is of note given the differences in IgA binding strength. In addition, our analysis of low-binding MAb variants demonstrated that dimeric IgA1 variants were 7 to 10 times more effective at agglutinating bacteria relative to monomeric IgA1 (Fig. 3C). Together, these data demonstrate a critical role of avidity in enhancing agglutination activity and, more importantly, suggest that how the antibody interacts sterically with the O-antigen may also be important to function.

As previously noted, motility inhibition is a critical property of V. cholerae neutralizing antibodies. Using a soft-agar motility assay, our analysis of antibody-mediated motility inhibition indicated that dimeric IgA1 variants of the low-binding MAbs were 7 to 10 times more potent than monomeric IgA1 (Fig. 3D). This demonstrates an important role for avidity driven enhanced cross-linking in mediating this function. Moreover, pentameric IgM and dIgA1 had comparable potencies, indicating that further enhancement of antibody mediated antigen cross-linking does not markedly enhance motility inhibition (Fig. 3D). In addition to these findings, we observed that isoforms derived from the two low-affinity MAbs had inhibitory potencies that were generally equivalent and, in some cases, modestly exceeded (2- to 4-fold) the immunoglobulin variants derived from both high-affinity antibodies (Fig. 3D). This is particularly notable in the context of the disparate binding potencies for all isoforms except for IgM (Fig. 3A), which incidentally, was modestly more effective in the context of variants derived from the two low-binding strength MAbs (Fig. 3D). The noncorrelative pattern (*r*^2^ = 0.03) of binding and motility inhibition between the high- and low-affinity MAbs is highlighted in Fig. S4, which depicts a linear regression analyses of the binding and motility inhibition titers described in Fig. 3A and D. These findings are similar in many respects to the agglutination analyses. Thus, this surprising result further underscores the hypothesis that the epitope and steric properties of the antigen-antibody interaction are critical to function.

10.1128/mBio.03679-20.4 FIG S4 No correlation between the binding potency and motility inhibition between the high- and low-affinity MAbs. The relationship between the binding and motility inhibiting properties of the high- and low-binding-derived antibodies was assessed. Values for binding represent the log_10_-transformed values of the antibody binding to O1-Ogawa O antigen, as determined by ELISA. Values for motility represent the log_10_-transformed values of the minimum effective MAb concentration that inhibited bacterial migration in a soft-agar motility inhibition assay. Linear regression analysis was performed on the transformed data, and the coefficient of determination (*r*^2^) was determined. Download FIG S4, PDF file, 0.02 MB. Copyright © 2021 Kauffman et al. 2021 Kauffman et al. https://creativecommons.org/licenses/by/4.0/ This content is distributed under the terms of the Creative Commons Attribution 4.0 International license.

### IgA2 variants have a functional potency comparable to that of IgA1 antibodies.

IgA1 is the dominant IgA subclass in the small intestine where antibody mediated immunity to cholera is likely to occur. Therefore, our study initially focused on the generation and characterization of antibodies in an IgA1 backbone. Nevertheless, IgA2 is inherently more resistant to IgA proteases in the gut that readily degrade IgA1 antibodies (29). Given the unique differences in tissue distribution and subclass-specific biochemical properties, we expanded our analyses to evaluate the binding, agglutination, and motility inhibitory properties of these MAbs expressed in a human IgA2 backbone (see Fig. S5). We observed that IgA2 antibodies had comparable O-antigen binding properties, though there was a suggestion that some may exhibit a 2- to 3-fold lower binding potency relative to IgA1 (see Fig. S5A). Agglutination analyses suggested that antibodies expressed as dimeric IgA2 were modestly more potent (2- to 4-fold) than IgA1. However, this difference was not observed when expressed as IgA2 monomers (see Fig. S5B). Lastly, IgA1 and IgA2 variants were not different in regards to bacterial motility inhibition potency (see Fig. S5C). Because we observed no difference in the functional capacity of IgA1 and IgA2, the enhanced resistance to proteolytic degradation seen in IgA2 antibodies may be a consideration in the development of strategies that aim to induce IgA subclass-specific immune responses or deliver MAb therapies or prophylactics.

10.1128/mBio.03679-20.5 FIG S5 IgA2 variants have comparable functional potency to IgA1 antibodies. (A) Binding to Ogawa O-antigen was determined by ELISA. Values represent the MAb concentration with signal three times the background. (B) Values represent the minimum effective MAb concentration that agglutinated bacteria in a microtiter agglutination assay. (C) Values represent the minimum effective MAb concentration that inhibited bacterial migration relative to a no-antibody control in a soft-agar motility inhibition assay. The dotted line marks the highest concentration of antibody evaluated (A = 20 nM, B = 50 nM, C = 50 nM). Values represent the mean of duplicate measurements and are representative of results from two independent experiments. Download FIG S5, PDF file, 0.1 MB. Copyright © 2021 Kauffman et al. 2021 Kauffman et al. https://creativecommons.org/licenses/by/4.0/ This content is distributed under the terms of the Creative Commons Attribution 4.0 International license.

### O-antigen-specific MAbs inhibit motility in a binary fashion, without affecting the speed of nonneutralized vibrios.

**FIG 4:**
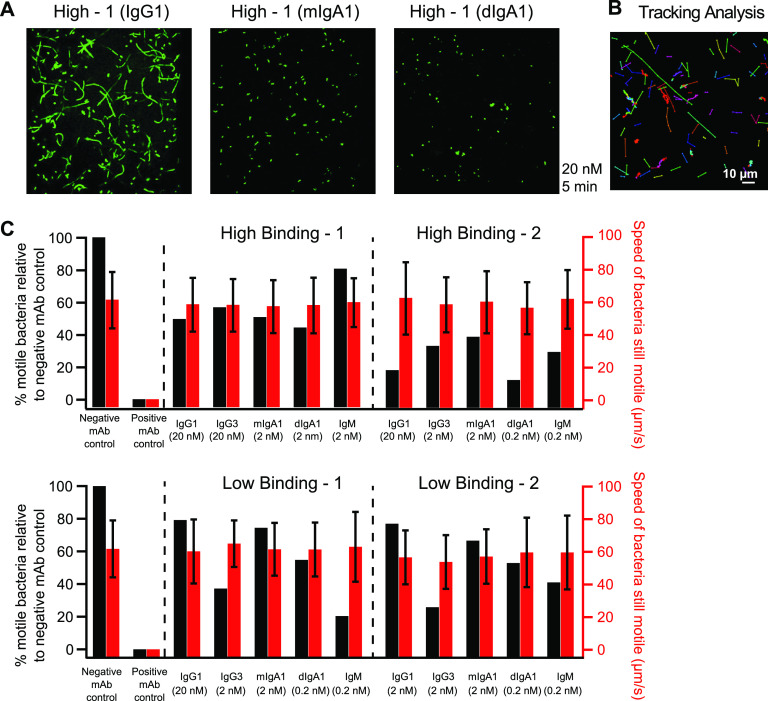
O-antigen-specific MAbs rapidly inhibit motility without affecting the speed of motile bacteria. The motion of a GFP-expressing V. cholerae O1-Ogawa strain was examined at 37°C using live-cell confocal microscopy 5 min after 20 nM antibody treatment. (A, left) Representative image depicting limited inhibition of motility induced by the IgG1 variant of MAb High-1. (Center) Gain of function by changing the isotype to IgA. (Right) Absence of motility in the presence of dimeric IgA. (B) Representative image of motility tracking analysis over 10 frames collected at 100-ms intervals. Individual bacteria are connected by colored lines. (C) Antibodies were titrated to a concentration (shown in parentheses) that partially inhibited the number of motile bacteria relative to a paired negative-control antibody. The percentages of motile bacteria (left *y*-axis) in MAb-treated samples are shown as black bars. Red bars represent the mean speeds (right *y*-axis) of the remaining motile bacteria, with overlaid bars indicating the standard deviations. EM4C04 (IgG1) was used as a negative control, while the IgM variant of MAb Low-1 was used as a positive control at 20 nM. Image series were collected for 5 s at 100-ms intervals. The data in panel A are representative of two independent experiments, while the data in panel C were collected in three separate experiments.

Soft-agar motility inhibition assays do not discern the relative effect of bacterial agglutination from direct antibody-mediated inhibition of flagellar motion. They are also unable to determine whether motility inhibition is a binary process whereby some bacteria are rendered immotile, while others are unaffected, or if it is a continuous process, in which antibodies are able to reduce the speed of individual vibrios. To investigate these questions, we used confocal microscopy to observe the effect of antibody treatment on the motility of a green fluorescent protein (GFP)-expressing V. cholerae O1-Ogawa strain (RT4273). Initial observations with MAb High-1 confirmed previous soft-agar migration analyses (Fig. 2), demonstrating that the IgG1 variant of this antibody is relatively ineffective. This was indicated by the presence of extended fluorescent tracks made by motile bacteria even in the presence of 20 nM antibody (∼3 μg/ml) (Fig. 4A, left). In contrast, changing the isotype to monomeric IgA1 resulted in a significant gain of function, in which complete arrest was observed 5 min post treatment at the same concentration (Fig. 4A, center). An equivalent phenotype was observed for the dimeric IgA1 variant of this antibody (Fig. 4A, right). Incidents of microagglutination consisting of 3 to 5 cells were observed in all samples, although this was more pronounced for the IgA-treated samples. Observing the arrest of an individual bacterium is challenging due to their speed and movement in and out of the plane of viewing. However, shortly after adding the antibody, we observed incidents of motility arrest in which the bacterium was isolated at the time motility ceased (data not shown). This is similar to observations made by other investigators (38, 39), indicating that antibody induced motility arrest can occur at the level of the individual bacterium in the absence of agglutination.

Next, we assessed if motility inhibition was primarily a binary process or a continuous one. This was accomplished using a novel image enhancement, segmentation, and tracking algorithm, which quantified the number of moving bacteria and determined their average speed using a series of time-lapse two-dimensional (2D) fluorescent images. An example of the tracking system image output is shown in Fig. 4B. In this panel, individual tracked objects are colored, and their positions at 100-ms intervals are connected by a line. Using this analysis, we found that the mean speed of motile bacteria in negative MAb isotype control samples was 62 μm/s (Fig. 4C, red bars; right *y*-axis). This is similar to the previously reported V. cholerae mean speed measured at 60 μm/s (40), thereby validating this approach. To assess the effect of antibody treatment on bacterial speed, we combined a mid-log-phase culture with a titrated amount of antibody to achieve a 20 to 80% reduction in the number of motile bacteria (Fig. 4C, black bars; left *y*-axis) relative to treatment with a negative isotype control MAb. This was necessary as adding too much MAb rapidly halted all bacterial motion, whereas adding too little resulted in no effect. Each experiment counted at least 25 motile bacteria in antibody treated samples (mean = 137, range = 26 to 437, standard deviation [SD] = 103) and 100 motile bacteria in control samples (mean = 290, range = 103 to 741, SD = 200). This analysis indicated that the MAb concentration necessary to render a portion of the sample immotile approximated the minimal effective concentration in soft-agar motility inhibition assays (Fig. 3). Importantly, using this quantitative live-cell tracking approach, we observed that the mean speed of the remaining motile bacteria was not significantly different from that of paired negative controls (*P* > 0.1; one-way analysis of variance [ANOVA]). This suggests that antibodies primarily inhibit V. cholerae motility in a binary manner.

### Evaluation of F(ab) fragments demonstrates that antigen cross-linking is important to binding potency and essential to motility inhibition.

**FIG 5:**
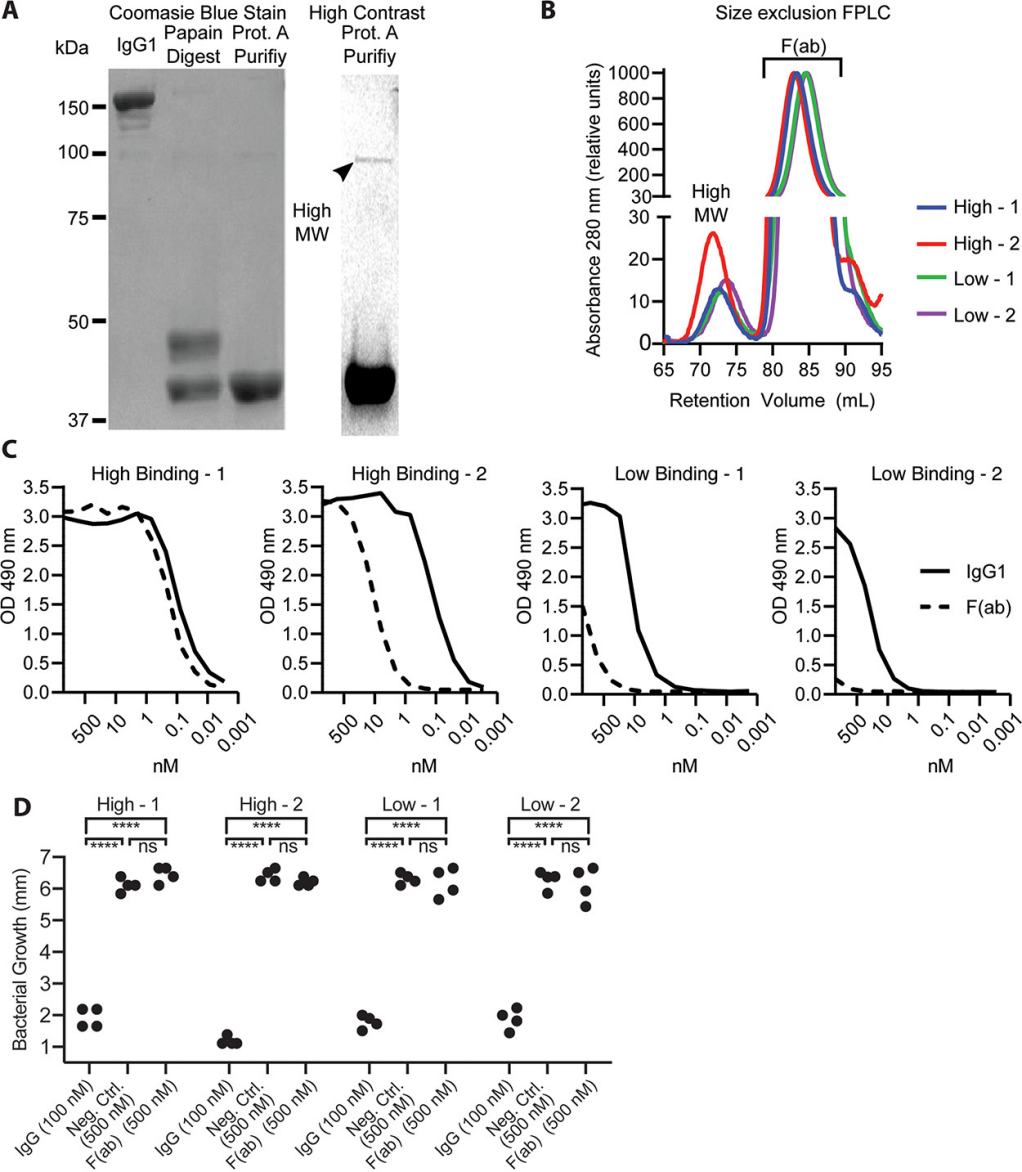
Antigen cross-linking is important to O-antigen binding potency and essential to motility inhibition. (A) Representative Coomassie blue-stained nonreducing SDS-PAGE gels of F(ab) fragment preparation from IgG1: before papain digestion (left), after papain digestion (center), and after protein A purification (right). A high-contrast version of the F(ab) post-protein A purification lane is shown as a separate image; the arrowhead indicates a rare ∼100-kDa protein. Each lane contains 4 μg of protein. (B) Chromatogram of 280 nm absorbance during F(ab) purification by SEC using a HiLoad 16/600 Superdex 200-pg column. Proteins eluted between 70 and 75 ml represent the high-molecular-weight (MW) species, while those eluted between 80 and 90 ml represent the F(ab) fraction. Absorbance values are normalized relative to the largest peak to account for loading differences. (C) Binding to Ogawa O-antigen was determined by ELISA for SEC purified F(ab)s (dashed black line) and undigested IgG1 antibody (solid black line). (D) Purified F(ab) fragments were tested at 500 nM in the soft-agar motility inhibition assay. Each assay included 500 nM EM4C04-IgG1 as a negative control and a positive control consisting of the IgG1 form of each antibody at 100 nM. Motility was assessed by measuring the diameter of bacterial growth after incubation at 30°C for 7 h. Significance between bracketed samples was determined by using one-way ANOVA. The *P* values were not significant (ns): 0.47 < *P* < 0.95. Data shown are from two independent experiments, with each sample tested in duplicate.

Lastly, we generated highly purified F(ab) fragments to investigate whether O-antigen cross-linking is essential to antibody mediated motility inhibition. In addition, we used monovalent F(ab)s to assess the impact of avidity on binding potency given our observations that modifying the isotype, subclass, and valency could affect binding (Fig. 3). F(ab)s were prepared from the IgG1 variant of each MAb by conventional papain digestion and purification (Fig. 5A). In addition to F(ab) fragments, this procedure generated a rare 100-kDa product, which was resistant to sequential protein A purification treatments and evident using high-contrast imaging in Coomassie blue-stained gels (Fig. 5A). SEC was used to resolve the two products and obtain highly pure F(ab) reagents (Fig. 5B). We were then able to conduct binding and motility inhibition studies using these highly pure reagents.

ELISA-based assays used to assess the contribution of engaging both IgG arms demonstrated that F(ab)s from MAbs High-2, Low-1, and Low-2 had a >100-fold reduction in binding potency relative to the parent IgG1 MAb (Fig. 5C, solid black line). These data demonstrate that multivalent binding is critical to the overall binding characteristics of these three antibodies. This is in stark contrast to MAb High-1 where F(ab)s had equivalent binding potency to the parent IgG1 (Fig. 5C). Next, after completing the previously described binding studies demonstrating that F(ab)s retain antigen binding, though at reduced levels relative to IgG1, we evaluated whether the highly pure F(ab) preparations would inhibit motility in the absence of cross-linking. This was performed using the soft-agar motility inhibition assay in which the semisolid agar was infused with F(ab)s at a high concentration (500 nM). Motility inhibition was assessed relative to a negative IgG1 isotype control at 500 nM and the bivalent IgG1 version of each antibody at 100 nM (Fig. 5D). Importantly, for all four antibodies, F(ab)s did not significantly (*P* > 0.5) inhibit bacterial motility, as evidenced by the equivalent diameter of the bacterial growth ring in F(ab)-treated and isotype control wells (Fig. 5D). This result is consistent with a critical role of O-antigen cross-linking in motility inhibition and suggests that epitope specificity determines whether an IgG molecule can engage both arms simultaneously when binding to LPS.

## DISCUSSION

While we anticipated that multimeric IgA and IgM would have enhanced binding properties due to their higher avidity, we were surprised by the extent to which antibody isotype and subclass affected binding and function. With regard to binding, we found that the low-binding MAbs, but not the high-binding MAbs, were exceedingly sensitive to changes in isotype and subclass. Increased binding potency of the IgG subclasses was generally associated with larger mean F(ab)-F(ab) binding angles (41). Of the four IgG subclasses, maximal binding of both low-binding MAbs was observed when they were expressed in an IgG3 background. In addition to having the largest mean F(ab)-F(ab) binding angle, IgG3 also has the greatest hinge-fold flexibility (41). Together, this likely facilitates antigen binding by both F(ab) arms. This is supported by our observation that monovalent F(ab) fragments derived from low-binding MAbs had a >100-fold reduction in binding potency relative to bivalent IgG1, indicating that avidity is indeed critical to their binding strength.

Another important finding from this study is the observation that changing the isotype and subclass could enhance the functional properties of all four MAbs. Moreover, we made the intriguing observation that variants derived from both low-binding MAbs generally had equivalent or moderately enhanced functional potency relative to variants derived from the two high-binding antibodies. This finding could have implications for rational vaccine design, as it suggests that the functional relevance of V. cholerae O-antigen-specific antibodies may be dependent on the epitope and steric interaction between the antibody and the bacterium and not only the binding potency. Thus, when evaluating the immunological response to disease and vaccination, agglutination and motility inhibition assays should be considered in addition to binding driven measurements such as ELISA. The ability of the vibriocidal assay to reflect both binding and antibody functionality, including agglutinating and antimotility activity, should be further explored. This study did not utilize X-ray crystallography to definitively determine the binding configuration of the complex formed between the antibody and the O-antigen; however, we previously reported that the low-binding MAbs possessed a higher degree of O1 serotype selectivity than the high-binding MAbs (14). Since the O-antigen structure of the Ogawa and Inaba serotypes differs solely by the structure of the terminal sugar moiety, it is intriguing to speculate that the low-binding MAbs primarily interact with the terminal sugar moiety and that the steric nature of this interaction is critical to their binding and remarkably strong functional properties. The structure of an O-antigen-antibody complex has been solved for at least one Ogawa-selective, mouse-derived, MAb termed S-20-4 (42). The crystal structure revealed that the antigen binding site formed a pocket that accommodated the terminal sugar. This positioned the antibody at the surface of the LPS layer orthogonal to the bacterial outer membrane. If similar interactions occur with the low-binding MAbs described here, then their enhanced activity may be due to the fact that their perpendicular position on the surface of the outer membrane facilitates cross-linking of LPS terminal sugar residues. In conjunction with our F(ab) fragment analyses demonstrating that antigen cross-linking is essential to motility inhibition, this would further help to explain why antibodies with greater F(ab)-F(ab) binding angles and avidity have enhanced binding and motility inhibiting properties.

A panel of antibodies with similar properties was described by Roche et al. in which the binding and agglutination characteristics of Francisella tularensis LPS O-antigen-specific MAbs were described (43). Importantly, MAbs that bound to internal, O-antigen side chain epitopes had very high affinities but poorly agglutinated bacteria. In contrast, antibody FB11, which bound to the terminal sugar moiety, had a much lower binding affinity, yet strongly agglutinated bacteria. Their study also demonstrated that internal epitope binding antibodies were markedly less dependent on avidity (43). Another study by the same group identified additional MAbs similar to FB11. The X-ray crystal structure of one of these MAbs (N62) showed that the antibody formed a pocket accommodating the terminal sugar similar to that of V. cholerae O-antigen-specific MAb S-20-4. Consequently, the antibody was well positioned to cross-link proximal antigens (44). We were unable to segregate our panel of antibodies into distinct terminal sugar and internal sugar O-antigen epitopes by competition ELISA (data not shown). Nevertheless, we observed that while MAbs High-1 and High-2 had similar binding affinities, MAb High-2 was indeed more potent in agglutination and motility inhibition assays. Thus, it is conceivable that the two high-binding MAbs interact differently with the O-antigen, which ultimately influences their ability to cross-link LPS molecules. While we did not experimentally demonstrate that MAb High-1 does not easily cross-link LPS, our F(ab) binding analyses showed that it retains high binding activity even without cross-linking. In contrast, MAb High-2 derives much of its high-binding strength from cross-linking. Ongoing biochemical and structural analyses aim to elucidate specifically how these MAbs interact with the O antigen and reveal the mechanisms by which these antibodies function.

IgM is more effective than IgG in its ability to activate the classical complement pathway. Without knowing the relative contribution of each isotype to the overall vibriocidal titer, a relatively low titer dominated by IgG antibodies may reflect a more mature, isotype-switched, response. Such a response could reflect a higher degree of protection than a response with a higher IgM dominant vibriocidal titer and may be better reflected in mucosal IgA responses not assessed by the vibriocidal assay. Our data support this possibility given the finding that monoclonal IgM was the most vibriocidal immunoglobulin isoform demonstrating a potency that was at least 20- to 200-fold more potent than IgG for the two high-binding MAbs, while having similar binding strength. Given our findings demonstrating the exceptional potency of the IgM isotype, coupling vibriocidal analyses to measures of total and specific serum IgG and IgM vibriocidal antibodies may be useful in dissecting the maturation of the immune response and its correlation to protection.

Antibody-mediated motility arrest at the level of the individual V. cholerae bacterium has been shown to occur independently of agglutination by multiple investigators using LPS-specific antibodies (21, 38, 39). This effect seems to be a key feature of LPS-specific antibodies. Other antigen targets, such as flagellin and OmpU, have not been demonstrated to effectively inhibit cholera motility or provide protection *in vivo* (45). Multiple models have been proposed to describe how these antibodies inhibit motility. For example, it has been suggested that antibody binding to the O-antigen may induce a stress response and subsequent signal cascade that arrests motility (39, 46). Motility could also be inhibited by cross-linking the flagella to the bacterial body; however, overcoming the force of the rotating flagella may be an obstacle to this model (39). Alternatively, while the flagella might not ultimately be cross-linked to the bacterium, these shear forces could cause significant damage to the flagella, flagellar sheath, or outer membrane. In addition to these models, it has been proposed that cross-linking LPS on the sheathed flagella could deform its structure and inhibit its function (38). Finally, analyses of O-antigen-specific antibodies that bound the enteric pathogen Cronobacter turicensis indicated that MAbs could generate micropores in the outer membrane, resulting in the neutralization of the membrane potential necessary for flagellar rotation (47). Although our study does not conclusively ascertain the mechanisms by which our panel of antibodies affect motility, we made several observations that are instructive.

Our live-cell tracking analyses indicated that, at partially neutralizing conditions, the speed of the remaining motile vibrios was not significantly affected. This signifies that motility inhibition is not a continuous process but rather a binary one whereby once a critical threshold of antibody binding is met, the bacteria are rapidly rendered immotile. Candidate models for a binary mechanism include cross-linking of the flagella to the main body, loss of the flagella, a stress response, and the neutralization of the sodium motive force following a disruption in outer membrane integrity by shear forces induced by flagellar binding. In support of a stress response model induced by direct binding, previous studies have indicated that motility can be inhibited by non-cross-linking F(ab) fragments generated from LPS-specific MAbs, ZAC-3 and 2D6 (39, 48). However, these findings were not supported in a subsequent study utilizing LPS-binding F(ab)s derived from polyclonal serum isolated from mice immunized with V. cholerae outer membrane vesicles (38) or from convalescent cholera patients (21).

Importantly, F(ab)s purified by SEC in this study also did not significantly affect motility at a concentration of 500 nM (25 μg/ml), which is comparable to the 15 μg/ml F(ab) concentration previously reported (39, 48). In contrast, motility was inhibited in assays using non-SEC purified F(ab)s from MAb High-2 (see Fig. S6). The high-molecular-weight protein component of the F(ab) preparation comprised approximately 3% of the total protein, had binding properties similar to bivalent IgG1, and may primarily have a structure similar to a bivalent F(ab′)_2_ (see Fig. S7). Therefore, assays performed at 500 nM total protein would contain an ∼7.5 nM concentration of this species, which is above the minimum effective motility inhibition concentration of High-2 IgG1. Taken together, these findings raise questions in regard to previous observations of F(ab)-induced motility inhibition and its support of a stress response model of motility inhibition. In contrast, our findings demonstrate that O-antigen cross-linking is essential to a binary mechanism of motility inhibition.

10.1128/mBio.03679-20.6 FIG S6 Pre-size exclusion F(ab) preparations derived from MAb High-2 inhibit motility. (A) Motility inhibition was assessed using the soft-agar migration assay. Purified F(ab) fragments were tested at concentrations of 500 and 100 nM. Each test plate included 500 nM EM4C04-IgG1 as a negative control and a 100 nM concentration of the IgG1 parent of each tested F(ab) as a positive control. Motility was assessed by measuring the diameter of bacterial growth after incubation at 30°C for 7 h. Values are expressed as a percentage of the growth ring diameter of paired negative controls, with the dotted line at 100% indicating equivalent growth to the negative control. Significance was determined using a one-way ANOVA compared to negative control values (**, *P* < 0.0001; *, *P* = 0.01). Values were obtained in two independent experiments with each sample tested in duplicate. (B) Live-cell confocal microscopy and time-lapsed tracking analysis was used to assess motility inhibition in a concentration dependent manner for MAb High-2 Pre-SEC F(ab) preparations. Bars represent the percentages of motile bacteria relative to the negative IgG1 control (EM4C04) at 500 nM. Antibody High-2 (IgG1) was used as a positive control at 100 nM. The data are from one assay and are representative of two independent experiments. Download FIG S6, PDF file, 0.1 MB. Copyright © 2021 Kauffman et al. 2021 Kauffman et al. https://creativecommons.org/licenses/by/4.0/ This content is distributed under the terms of the Creative Commons Attribution 4.0 International license.

10.1128/mBio.03679-20.7 FIG S7 The high-molecular-weight protein species in conventional F(ab) preparations has binding properties similar to bivalent IgG1 and gel migration characteristics comparable to F(ab′)_2_ and F(ab) aggregates. (A) Protein sample absorbance at 280 nm during F(ab) purification by SEC. The high-molecular-weight (MW) species is approximately 1.5 to 3% the abundance of the F(ab) population for all four antibodies. (B) Binding to Ogawa O-antigen was determined by ELISA for SEC-purified F(ab)s (dashed black line), undigested IgG1 antibody (solid black line), and the purified, 100-kDa, high-MW protein species (solid red line). Due to the limited amount of purified high-MW protein (solid red line), binding was measured starting at a reduced concentration of 10 nM. (C) Representative Western blot analysis of the purified F(ab) and high-MW proteins relative to F(ab′)_2_ generated by IdeZ protease digestion of IgG1. Protein was detected using anti-human IgG(H+L)-AF790 antibody using a Li-COR Odyssey imager. Download FIG S7, PDF file, 1.0 MB. Copyright © 2021 Kauffman et al. 2021 Kauffman et al. https://creativecommons.org/licenses/by/4.0/ This content is distributed under the terms of the Creative Commons Attribution 4.0 International license.

In conclusion, understanding the impact of isotype and subclass on the functional properties of human-derived O-antigen-specific antibodies will significantly improve our understanding of what types of adaptive immune responses are the most beneficial. This will ultimately inform the development of more effective vaccines and therapies. To that end, findings from this study support the importance of antibodies directed against the O-antigen component of LPS. Our observation that variants derived from the low-affinity antibodies generally had equivalent or enhanced functional activity relative to variants derived from the high-affinity antibodies also suggests that how an antibody binds the O-antigen may strongly influence functional potency. Given the serotype selective properties of the low-affinity antibodies, it is intriguing to speculate that antibodies targeting the terminal sugar moiety may have superior functional activity. Future studies utilizing high-resolution imaging, component labeling techniques, and structural analyses will help elucidate the mechanisms that mediate their enhanced agglutination and motility inhibition properties. In addition to our surprising findings regarding the low-affinity antibodies, we found that monomeric IgA was exceedingly potent in regard to bacterial agglutination and motility inhibition for all four antibodies relative to IgG1. Studies have reported that glycans in the IgA hinge region may augment nonspecific binding and agglutination and can induce a T-shaped conformation (49). In the current context, this would enhance the binding of widely spaced antigens and facilitate interactions between the flagella and the main body (50, 51). Thus, in addition to the steric nature of the O-antigen–F(ab) interaction, the inherent properties of IgA may also make a significant contribution to functional potency. Given the enhanced functional properties of IgA variants and the fact that IgA is readily transported into the intestinal lumen, our data suggest that future vaccine designs should promote the generation and maintenance of intestinal B cells that secrete IgA, as well as IgM, that interacts with the O-antigen in a manner similar to the low-binding MAbs described here.

## MATERIALS AND METHODS

### Subcloning variable domains into isotype and subclass expression vectors.

The heavy chain variable domain was amplified by PCR using 1 ng of a previously generated IgG1 expression vector (14) and subcloned into the heavy-chain expression vectors IgG2, IgG3, IgG4 (a gift from Hedda Wadermann, Max Plank Institute), IgA1, IgA2 (52) (a gift from Hugo Mouquet, Institut Pasteur), and IgM (53) (a gift from Lynn Dustin, University of Oxford). Amplification of the variable domain was performed using a high-fidelity DNA polymerase Phusion (NEB) in HF buffer according to the manufacturer’s recommendations. The forward primer AgeI-Ig Forward (5′-CCTTTTTCTAGTAGCAACTGCAACCGGTGTAC-3′) and the reverse primer XhoI/SalI-Ig Reverse (5′-CTTGGTCGACGCgctcGAGACGGTGACC-3′) were used at a final concentration of 500 nM. Underlined sequences denote restriction sites AgeI, SalI, and XhoI, respectively. To allow for sequences to be cloned into the IgM expression vector, the terminal four nucleotides of the J-chain “CTCA” were mutated by primer induced mutagenesis to “GAGC,” generating an XhoI restriction site without modifying the antibody amino acid sequence. The mutagenesis site is denoted by lowercase letters in the reverse primer. The reaction conditions were 98°C for 1 min, 25 cycles of 72°C for 30 s and 98°C for 10 s, a final extension 72°C for 5 min.

Following PCR, amplicons were purified using QIAquick PCR purification columns. For subcloning into IgG and IgA expression vectors, 10 ng of each amplicon was digested with AgeI-HF, SalI-HF, and DpnI in CutSmart buffer (NEB). To generate IgM vectors, amplicons were digested with XhoI instead of SalI-HF. Restriction digests were performed in a volume of 10 μl at 37°C for 30 min, followed by heat inactivation at 70°C for 20 min. Digested amplicons were ligated to heavy-chain vectors that had been appropriately cut and dephosphorylated. Ligations were performed overnight at 16°C with 10 ng of vector, 2 U of T4-DNA ligase (NEB), 1 mM ATP (NEB), and 1× CutSmart buffer in a 15-μl final reaction volume.

Ligations were chemically transformed into XL-10 gold ultracompetent Escherichia coli (Agilent) according to the manufacturer’s recommendations. Transformants were selected on Luria broth (LB)-ampicillin (100 μg/ml) agar plates. Individual colonies were grown overnight in LB-ampicillin broth, and plasmids were isolated using a QIAprep spin miniprep kit. Inserts were sequenced using the SP6 sequencing primer to confirm the identity of the cloned variable domain and the isotype and subclass of the vector.

### MAb expression and purification.

All MAbs were expressed by transient transfection using suspension expi293F cells (Thermo Fisher) cultured in expi293 expression media according to the manufacturer’s recommendations. Monomeric IgG and IgA transfections were performed using a 1:2 molar ratio of heavy- and light-chain expression vectors. For expression of dimeric IgA and pentameric IgM, transfections were performed using a 1:1:1 molar ratio of the heavy chain, light chain, and a third J-chain expression vector (gift of Hugo Mouquet). Supernatants were harvested 5 to 6 days after transfection. Supernatants were clarified by centrifugation at 3,000 × *g* for 5 min, followed by syringe filtration using 0.45-μm PES filters. IgG antibodies were purified by affinity chromatography using Protein G-Sepharose beads (Thermo Fisher). IgA antibodies were purified by affinity chromatography using IgA MAb select beads (Thermo Fisher). Monomeric IgG and IgA were purified by incubating supernatants and purification beads overnight at 4°C in a slowly rotating inversion mixer. Beads were sequentially washed in 2-ml protein centrifuge columns (Pierce) with 4 ml of Dulbecco phosphate-buffered saline (DPBS), 4 ml of 1 M NaCl, and 4 ml of DPBS. Antibodies were eluted with 4 ml of 0.1 M glycine (pH 2.7 [IgG] or pH 3.0 [IgA]) into 30-kDa protein centrifugation concentrators containing 260 μl (IgG) or 300 μl (IgA) of 1 M Tris-Cl (pH 9.0). Two buffer exchanges were performed with DPBS, and antibodies were suspended at a concentration between 0.5 and 2 mg/ml.

IgA transfections containing the J chain generated a mixed population of monomeric and dimeric antibodies, with each species comprising between 40 and 60% of the total antibody population. After initial binding chromatography purification of IgA, dimeric IgA was separated from monomeric IgA by gel filtration. Gel filtration was performed using a HiLoad 16/600 Superdex 200-pg column (GE Healthcare) on an AKTA Purifier 10 running Unicorn Software version 5.1 according to the manufacturer’s recommendations. Samples were eluted in PBS at a flow rate of 1 ml/min. Fractions with a retention volume between 50 and 55 ml, corresponding to dimeric IgA, were pooled and concentrated using a 30-kDa protein centrifugation concentrator to a concentration of 1 to 2 mg/ml.

Pentameric IgM was purified from supernatants by first concentrating the sample using a 100-kDa centrifugal filter. IgM was further purified by SEC using a HiLoad 16/600 Superdex 200-pg column. Approximately 4 ml of IgM containing supernatant was injected onto the column and eluted in PBS at a flow rate of 1 ml/min. The first 5-ml fraction eluted after the column void volume (∼40 ml) was collected. This fraction corresponds to proteins >600 kDa and predominantly contained pentameric IgM. The IgM-containing fraction was concentrated using a 30-kDa protein centrifugation concentrator to a protein concentration of 1 to 3 mg/ml. For all antibodies, sodium azide was added as a preservative at a final concentration of 0.05%.

Antibody concentration was determined by measuring the absorbance value (i.e., the optical density at 280 nm) using a Thermo Scientific NanoDrop 2000 spectrophotometer. Previously reported extinction coefficients for each isotype were used to determine protein concentrations (35). Finally, we used the average molecular weight of each isotype and subclass to calculate the molarity of each antibody stock solution (54, 55).

Antibodies were evaluated for purity and integrity by SDS-PAGE analysis. Reducing SDS-PAGE analysis was performed by combining 3 μg of antibody diluted in PBS with an equal volume of 2× Laemmli sample buffer (Bio-Rad) containing 5% 2-mercaptoethanol. Samples were heated at 95°C for 10 min and resolved on a 10% Mini-Protean TGX gel (Bio-Rad) in Tris-glycine-SDS running buffer. Nonreducing SDS-PAGE analysis of IgG antibodies was performed as described for reducing SDS-PAGE analysis except that 2-mercaptoethanol was omitted from the loading dye and samples were heated at 70°C for 10 min. For monomeric and dimeric IgA antibodies, samples were mixed with NuPAGE 4× LDS sample buffer and resolved on a NuPAGE 4 to 12% Bis-Tris Gel (Invitrogen) with MOPS-SDS running buffer. IgM samples were combined with NuPAGE 4× LDS sample buffer and resolved on a Native PAGE 3 to 12% Bis-Tris gel (Invitrogen) with Tris-acetate-SDS running buffer. Gels were stained in Coomassie blue staining solution (50% methanol, 10% acetic acid, 0.1% [wt/vol] Coomassie blue R-250) by microwaving the gel in 50 ml of staining solution for 1 to 2 min. Gels were destained by two to three rounds of boiling in 1 liter of deionized water for 10 to 15 min per cycle. Imaging was performed using a Bio-Rad Gel Doc XR+ system. Densitometry analysis was performed with ImageLab version 6.0 (Bio-Rad). SDS-PAGE and SEC were used to confirm that multivalent structure of dimeric IgA and IgM antibodies were stable during the course of experimental analyses and that monomeric IgA MAbs did not spontaneously form dimeric isoforms.

### Enzyme-linked immunosorbent assays.

V. cholerae O1-Ogawa O-specific polysaccharide core (OSP) was purified and conjugated to BSA as previously described (56, 57) from LPS extracted from V. cholerae strain X25049 by hot phenol-water extraction, followed by enzymatic treatment (56). OSP was coated at ambient temperature overnight on Nunc MaxiSorp plates at a concentration of 1 μg/ml in DPBS. Plates were blocked for 2 h at 37°C in PBS–1% BSA. MAbs were serially diluted 1:4 in antibody dilution buffer (ADB; PBS-1% BSA, 0.05% Tween 20) starting at a concentration of 20 nM. Then, 100 μl of each dilution was added to blocked plates, followed by incubation for 2 h at 37°C. In assays measuring relative binding differences between isotypes, 100 μl of horseradish peroxidase (HRP)-conjugated goat F(ab′)_2_ goat anti-human lambda or kappa secondary antibody (Southern Biotech) diluted 1:1,000 in ADB was added. Assays measuring binding differences only between IgG1 antibodies used HRP-conjugated F(ab′)_2_ goat anti-human IgG Fcγ antibody (Jackson ImmunoResearch) diluted 1:5,000 in ADB. Intermediate washes consisted of PBS–0.05% Tween (3×) and PBS (1×). Development was performed using 0.4 mg/ml *o*-phenylenediamine (OPD) in 0.05 M phosphate-citrate buffer (pH 5.0), supplemented with 0.012% hydrogen peroxide before use. Reactions were stopped with 1 M hydrochloric acid, and the absorbance was measured at 490 nm.

### Generation of F(ab)s.

F(ab) fragments were generated from IgG1 MAbs by papain digestion using a Pierce F(ab) preparation kit (Thermo Fisher) according to manufacturer’s instructions. After protein A purification, F(ab)s were further purified by SEC using a HiLoad 16/600 Superdex 200-pg column at 1 ml/min in PBS. Fractions with retention volumes of 70 to 75 ml and 80 to 90 ml, corresponding to the high- and low-molecular-weight F(ab) species, respectively, were pooled and concentrated with a 10-kDa centrifugal filter (Amicon, EMD Millipore). To assess F(ab) purity and composition, 200 ng of samples were prepared in reducing (2.5% β-mercaptoethanol) and nonreducing sample loading buffer and heated for 10 min at 95°C. Samples were then resolved by SDS-PAGE using Any kD Mini-Protean TGX precast gels (Bio-Rad). Proteins were blotted onto 0.45-μm nitrocellulose membranes at 100 V for 1 h in Towbin buffer. Membranes were blocked with Intercept TBS blocking buffer (LI-COR Biosciences). Protein was detected with a directly labeled anti-human IgG (H+L)-AF790 antibody (Jackson ImmunoResearch) and, after drying, the membrane was imaged using a LI-COR Odyssey CLX infrared imager. Washes between each step consisted of four 5-min washes in TBS–0.1% Tween 20.

### Vibriocidal and agglutination assays.

Assays were performed using V. cholerae strain O1-Ogawa (X25049). Vibriocidal assays were essentially conducted as previously described (58). In this study, MAbs were diluted 2-fold in 0.9% saline solution starting at 20 to 40 nM. Vibriocidal EC_50_ values were determined as the concentration of MAb that effected a 50% reduction in the average culture turbidity (OD_595_) of medium-only controls. Assays utilizing inactivated guinea pig complement were performed as described above but utilized complement that had first been heated for 30 min at 56°C before use. Minimum agglutinating titers were determined by diluting MAbs 2-fold in PBS starting at a concentration of 100 nM. Bacteria were prepared by growing each culture to mid-log phase (2 to 3 h) in bovine heart infusion media. Bacteria were then pelleted at 3,000 × *g* for 10 min and washed twice in PBS. Immediately before use, bacteria were normalized such that a 1:10 dilution of the sample had an OD_595_ of 0.3 ± 0.02 relative to PBS. For each antibody, duplicate measurements were performed in which 25 μl of the bacterial sample was combined with an equal volume of diluted MAbs added to a V-bottom microtiter plate. Plates were sealed with an adhesive film, briefly centrifuged to concentrate contents at the bottom of the well (20 × *g*, 15 s) and then incubated for 20 to 24 h at 4°C to allow nonagglutinated bacteria to form a “button” at the bottom of the well. Plates were imaged using a UV imaging system (ChemiDoc; Bio-Rad). Agglutination titers were recorded as the last dilution where bacteria were visibly agglutinated.

### Soft-agar motility inhibition assays.

Antibody-mediated inhibition of motility was measured using an antibody-infused soft agar bacterial migration assay. Assays were performed in 24-well plates containing 500 μl of LB–0.3% agar. Wells were prepared by performing 2-fold serial dilutions of antibodies in LB media starting at a concentration of 100 nM. Equal volumes of diluted antibody and 45°C, 0.6% molten LB agar (Fisher; catalog no. BP1423) were combined and mixed in a microplate mixer. Plates were allowed to rest overnight at ambient temperature. Wells were stab inoculated at the top of the well with an inoculation needle dipped in a mid-log-phase culture of V. cholerae O1-Ogawa (X25049). Plates were then incubated at 30°C for 7 to 8 h until bacterial growth in no-antibody control wells reached the well’s midpoint. The minimum effective concentration of antibody was measured as the lowest concentration of antibody that was able to retard bacterial migration relative to no-antibody control wells. For experiments utilizing F(ab) fragments, wells were stab inoculated in the center of the well, and the diameter of bacterial growth was measured after 7 h. Assays were performed with duplicate measurements tested on separate plates.

### Live-cell *V. cholerae* motility tracking.

The motion of the free-swimming cells after treatment with isotype and subclass antibody variants was examined by live-cell microscopy using a Zeiss Axio spinning-disk Observer Z1 microscope equipped with a 37°C heating chamber. A mid-log-phase culture of the GFP-expressing V. cholerae O1-Ogawa strain RT4273, derived from classical strain O395 (a gift from Ron Taylor’s laboratory, Dartmouth) was used at a concentration of approximately 1 × 10^8^ CFU/ml (OD = 1). This culture was prepared by subculturing bacteria from an overnight streak plate grown for 2 to 3 h in Luria broth (LB) with shaking at 250 rpm. Equal volumes of bacterial culture and antibody diluted in LB were gently mixed and then loaded in a u-Slide VI 0.4 Uncoated (Ibidi) chamber slide for direct imaging. Bacteria were detected with a 488-nm laser. Images were collected every 100 ms for 5 s. For each experiment, a minimum of 25 cells were analyzed for speed, vector direction, and general motility characteristics. The percentage reduction of the number of motile bacteria was determined relative to a negative influenza HA-specific IgG1 control antibody (EM4C04) at a concentration of 20 nM on each six-chamber slide in order to account for longitudinal differences in the absolute number of motile bacteria due to factors such as cell division during experimentation.

The motility characteristics of bacteria were obtained by our tracking algorithm that detected high-intensity bacteria and tracked their movements in a fluorescent image sequence. The local adaptive threshold algorithm was used to binarize the first frame of the image sequence, generating a set of foreground objects with their spatial locations and morphometry features as the initial state components for bacteria representation. In each of the following frames from an image sequence, it was first processed in the same way as the first image frame for a set of “true states.” Next, a set of “particles,” i.e., educated bacterium state guesses, was generated by adding random perturbations to the original states. The weights of the resulting particles were computed by comparisons to the binary image frame after segmentation. The weighted average of all particles of a bacteria state was treated as its estimated state and compared to the true states of all bacteria in the current image frame. With the estimated state of each bacterium, we computed pairwise similarities between the estimated state and each true state. If the best similarity was sufficiently good, the true state associated with the best similarity was selected as the state updates for each bacterium. Otherwise, this bacterium was considered moving out of the image focal plane or scope of view. In addition, those with unselected true states were considered newly emerging bacteria. By repeating this procedure, the trajectories of bacteria were obtained by the updated spatial state components with stepwise state evolutions. With these bacterial spatial trajectories and the known time interval between adjacent image frames, we computed the derived spatial speed for each bacterium. To distinguish motile and stationary bacteria, we set the speed cutoff value as 5 pixels, about 1% of the image frame size. Software to implement the image analysis is available upon request.

### Statistical tests.

Graphs and statistical tests were performed using GraphPad Prism software version 8.0. Either a one-way or a two-way ANOVA was used to determine statistical significance. Information about the statistics used for each experiment, including sample size, experimental method, and specific statistic test employed, can be found in the relevant results section or figure legend.
