# Supplementary figures and images for: Impact of Immunoglobulin Isotype and Epitope on the Functional Properties of Vibrio cholerae O-Specific Polysaccharide-Specific Monoclonal Antibodies
Source: mBio. 2021 Apr 20;12(2):e03679-20. doi: 10.1128/mBio.03679-20 (PMC8092325; doi:10.1128/mBio.03679-20)

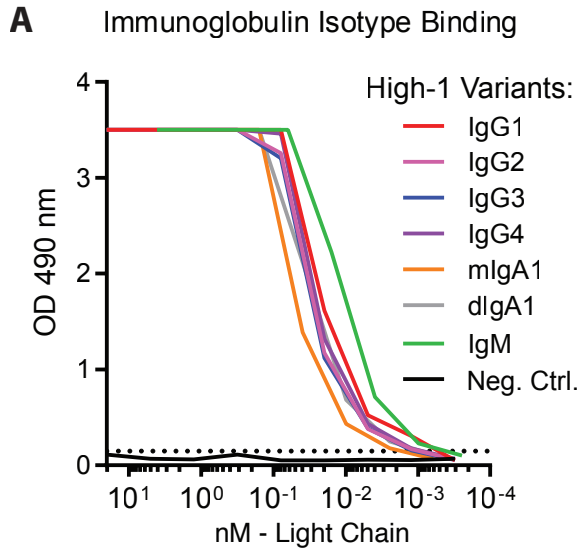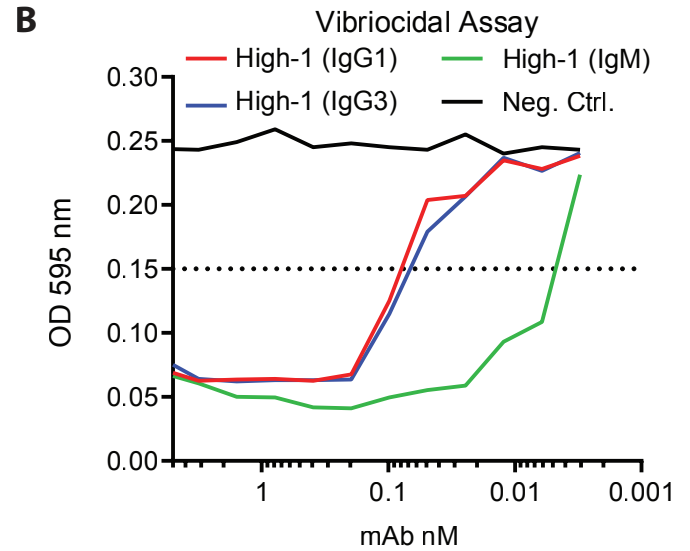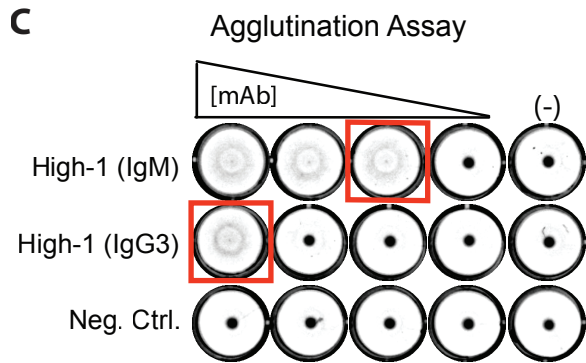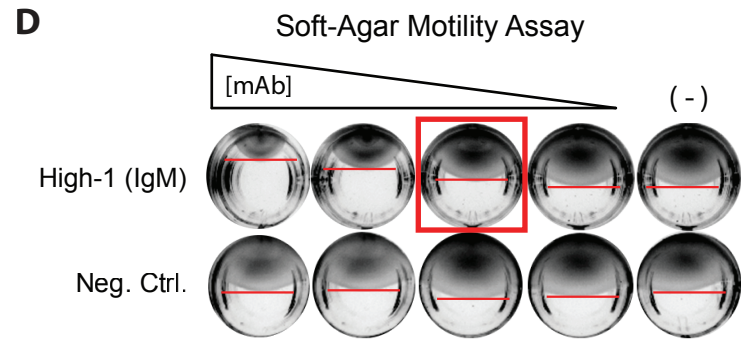

Supplement: FIG S1 [file mBio.03679-20-sf001.pdf]

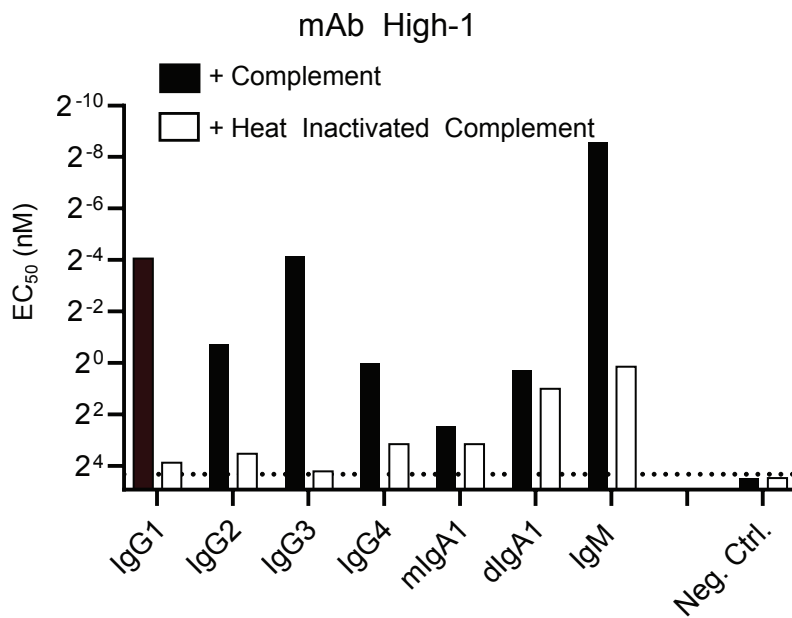

Supplement: FIG S2 [file mBio.03679-20-sf002.pdf]

Supplemental Figure S3

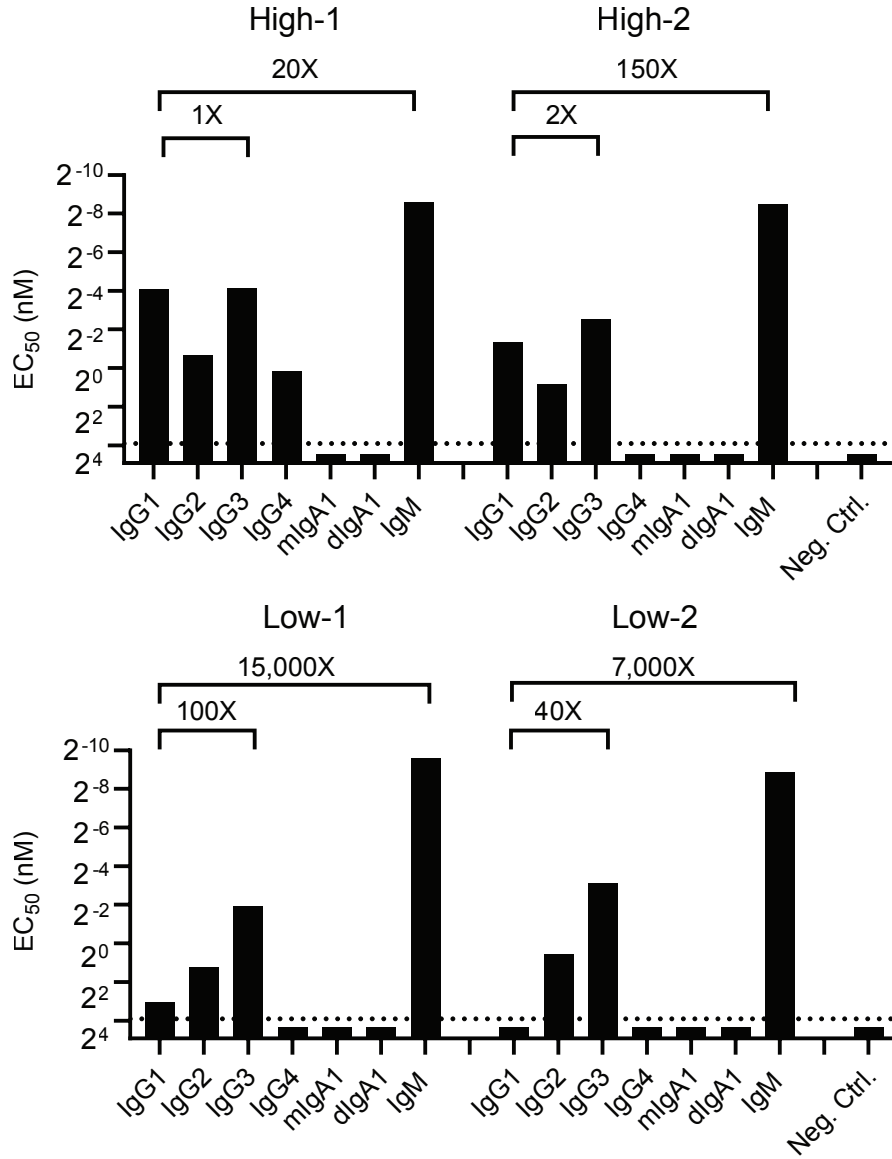

Supplement: FIG S3 [file mBio.03679-20-sf003.pdf]

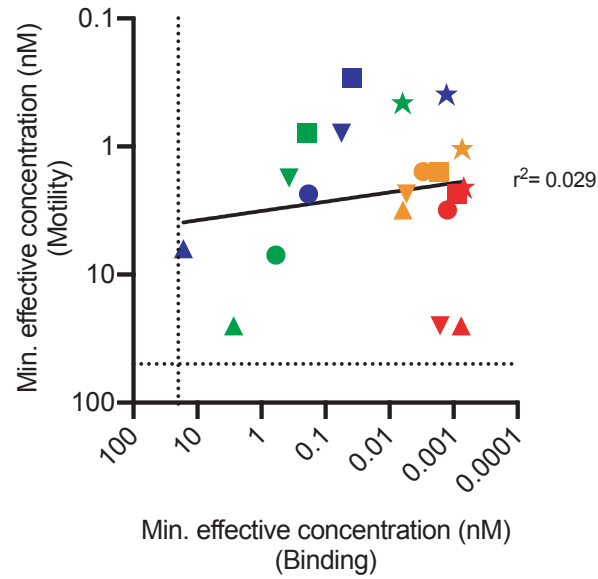

- |                        |         |
|------------------------|---------|
| ■ High affinity mAb #1 | ▲ IgG1  |
| ■ High affinity mAb #2 | ▼ IgG3  |
| ■ Low affinity mAb #1  | ● mIgA1 |
| ■ Low affinity mAb #2  | ■ dIgA1 |
|                        | ★ IgM   |

Supplement: FIG S4 [file mBio.03679-20-sf004.pdf]

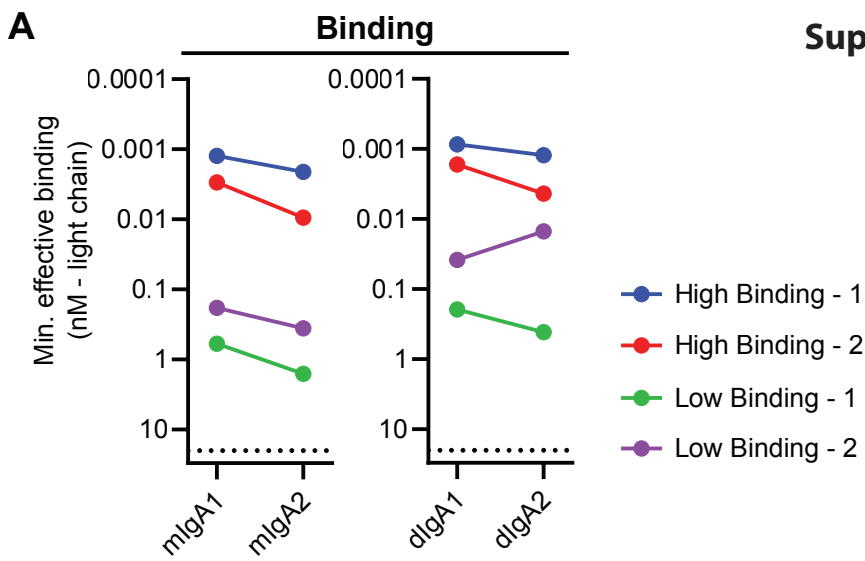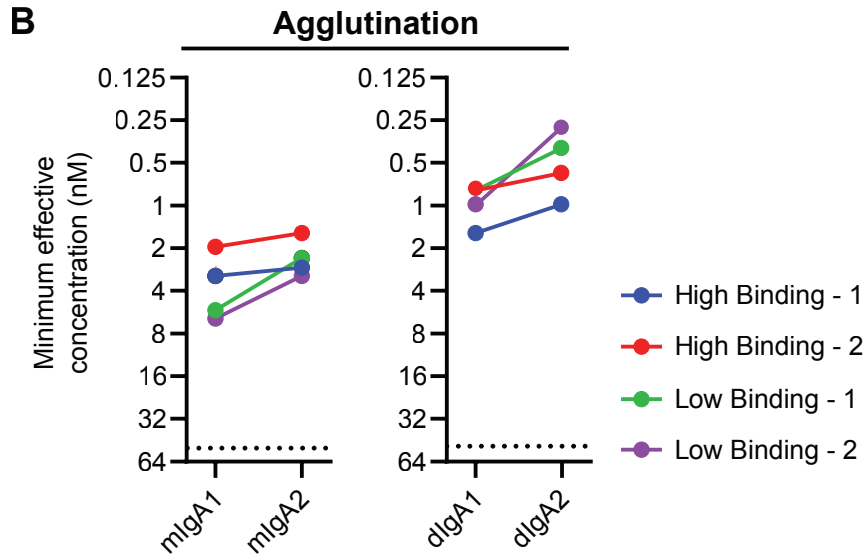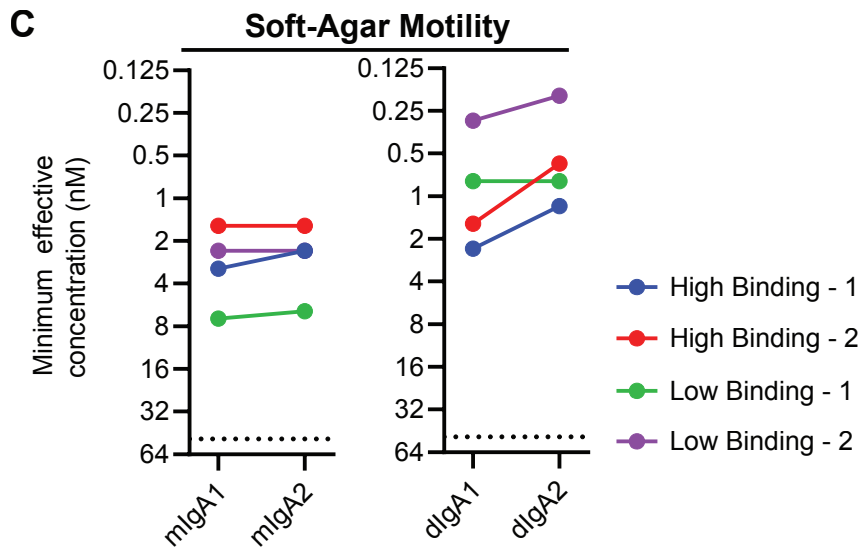

Supplement: FIG S5 [file mBio.03679-20-sf005.pdf]

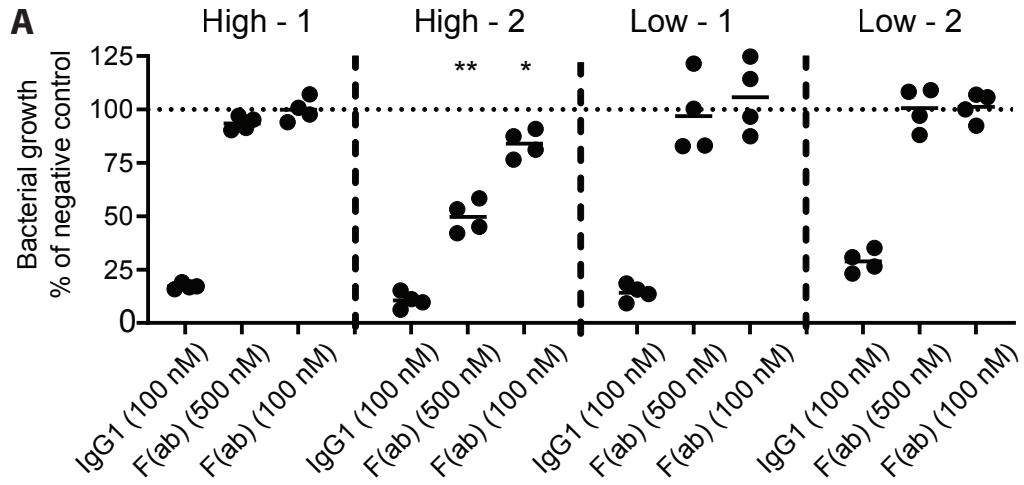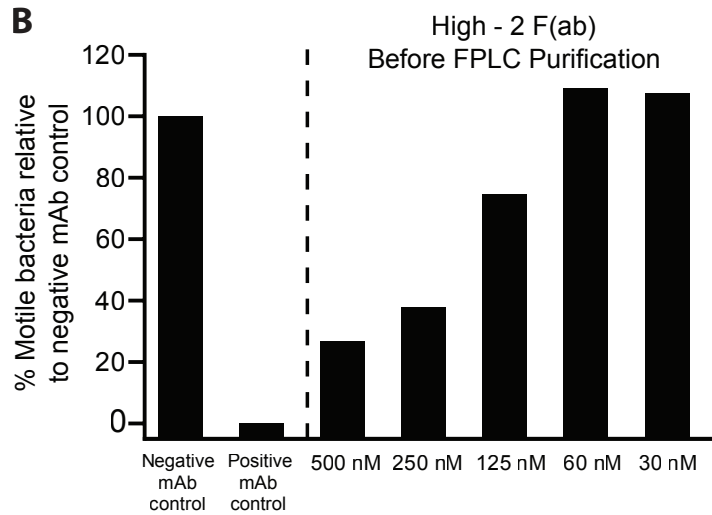

Supplement: FIG S6 [file mBio.03679-20-sf006.pdf]

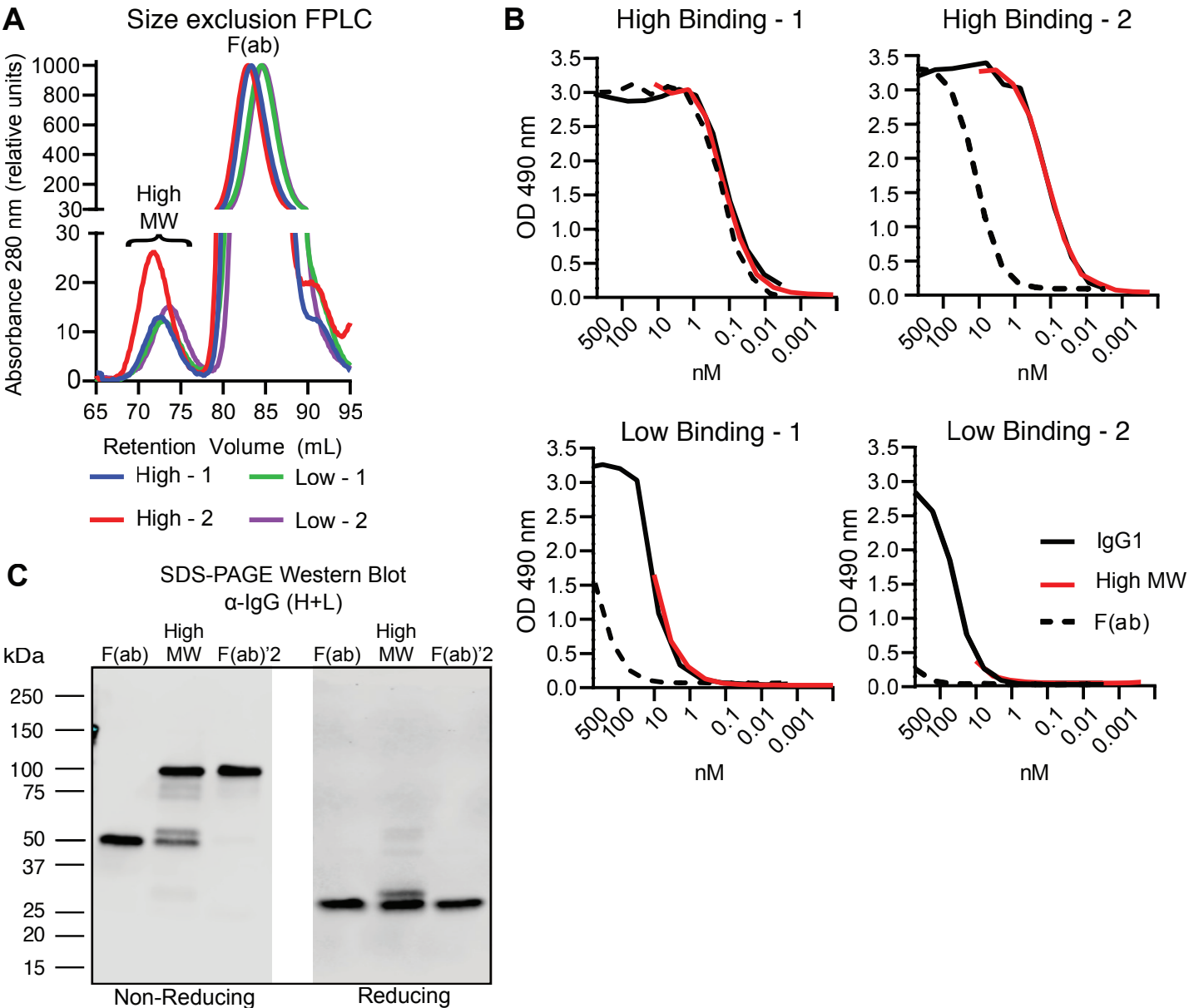

Supplement: FIG S7 [file mBio.03679-20-sf007.pdf]
